# Efficacy and safety of thymosin α1 combined with chemotherapy for gastric cancer: a systematic review and meta-analysis of randomized controlled trials

**DOI:** 10.3389/fimmu.2026.1884797

**Published:** 2026-09-18

**Authors:** Zekun Wang, Tao Sun, Haoran Qu, Ruping Zhao, Xishen Shan, Yu Liu, Yuling Zheng

**Affiliations:** 1 Department of Oncology, The First Affiliated Hospital of Henan University of Chinese Medicine, Zhengzhou, China; 2 Henan University of Chinese Medicine, Zhengzhou, China; 3 Paediatric Hospital, The First Affiliated Hospital of Henan University of Chinese Medicine, Zhengzhou, China

**Keywords:** chemotherapy, gastric cancer, grade, immunomodulation, thymosin α1, TSA

## Abstract

**Objective:**

To systematically evaluate the efficacy and safety of thymosin α1 combined with chemotherapy in the treatment of gastric cancer.

**Methods:**

A systematic search of 8 electronic databases was conducted to identify relevant RCTs. Meta-analysis was performed using Stata 18.0. Meta-regression, subgroup analyses, sensitivity analyses, and publication bias analyses were conducted to further examine results, and reliability of findings was assessed using TSA and GRADE.

**Results:**

20 RCTs involving 1,704 patients were included. Meta-analysis results showed that, thymosin α1 combined with chemotherapy was associated with higher ORR (RR = 1.39), DCR (RR = 1.16), and KPS scores (WMD = 8.27). Regarding immune function, combination therapy resulted in higher CD3^+^% (WMD = 7.73), CD4^+^% (WMD = 6.87), CD4^+^/CD8^+^ (WMD = 0.42), and NK cell (WMD = 4.73). Regarding tumor markers, combination therapy resulted in lower levels of CEA (WMD = −5.48) and CA199 (WMD = −9.06). Regarding inflammatory factors, combination therapy may reduce levels of MMP-2, MMP-9, IL-4, and IL-10, while increasing levels of IFN-γ and TNF-α. Regarding safety, combination therapy was associated with reductions in gastrointestinal reactions (RR = 0.63), myelosuppression (RR = 0.60), abnormal liver function (RR = 0.54), and neurotoxicity (RR = 0.47). However, included RCTs did not systematically report long-term survival outcomes, and their methodological quality was limited; and GRADE indicated that quality of evidence for outcomes ranged from very low to moderate.

**Conclusion:**

The combination of thymosin α1 with chemotherapy is associated with improved short-term efficacy, enhanced immune function, improved quality of life, and a reduced incidence of adverse reactions in patients with gastric cancer, indicating good potential for clinical application. However, it remains unclear whether this approach can provide long-term benefits for patients with gastric cancer. Furthermore, given that studies included were all small-sample studies conducted in China and were of limited quality, certainty of evidence is low; these conclusions require further validation through additional high-quality studies.

**Systematic Review Registration:**

https://www.crd.york.ac.uk/PROSPERO/, Identifier CRD420261282679.

## 1 Introduction

Gastric cancer is a common malignant tumor of the digestive system worldwide, ranking fifth in incidence and fourth in mortality among all malignant tumors, posing a significant burden on public health (1). Due to mild early symptoms and low screening rates, most patients with gastric cancer are already at a locally advanced stage or have distant metastases at the time of diagnosis, thereby losing the opportunity for curative surgery (2). Even after curative resection, the postoperative recurrence rate remains high (3, 4). Statistics show that the five-year survival rate for patients with advanced gastric cancer is less than 15% (5), highlighting an urgent need for more effective comprehensive treatment strategies to improve patient prognosis. Chemotherapy is one of the primary treatment modalities for locally advanced and metastatic gastric cancer, often used as neoadjuvant therapy before surgery, adjuvant therapy after surgery, or as a first-line treatment regimen for advanced disease. Chemotherapy regimens based on fluoropyrimidine-platinum combinations, such as XELOX (capecitabine plus oxaliplatin) and SOX (tegafur plus oxaliplatin), have been recommended by multiple clinical guidelines as standard treatments for gastric cancer (6–8). However, the efficacy of chemotherapy varies among individuals and is often accompanied by significant adverse effects, such as bone marrow suppression, gastrointestinal reactions, and neurotoxicity, which limit its clinical efficacy and patient tolerability (9–11). Therefore, how to improve the efficacy of chemotherapy while controlling adverse reactions is a key focus of current research in gastric cancer treatment.

In recent years, the role of immunomodulators in cancer treatment has gradually gained attention (12). Among these, thymosin α1 is an immunologically active peptide composed of 28 amino acids, initially used to treat viral hepatitis and immunodeficiency disorders (13). Recent studies have shown that thymosin α1 can restore and enhance the body’s antitumor immune response through multiple mechanisms, including promoting T-cell differentiation and maturation, enhancing natural killer cell activity, and regulating cytokine release (14, 15). Furthermore, basic research suggests that thymosin α1 may also exert a synergistic effect on chemotherapy by modulating the tumor microenvironment and inhibiting immune evasion (16–19). Preliminary clinical studies have also shown that the combination of thymosin α1 and chemotherapy may improve patients’ quality of life and alleviate chemotherapy-related immunosuppression in various solid tumors; however, its efficacy and safety in the treatment of gastric cancer remain controversial (20–23).

To date, several randomized controlled trials (RCTs) have investigated the efficacy of thymosin α1 in combination with chemotherapy in patients with gastric cancer (24–27). Some studies suggest that this combination therapy can improve objective response rates (ORR) and quality of life while reducing the incidence of chemotherapy-related adverse reactions; however, other studies have not observed significant clinical benefits. These studies exhibit considerable heterogeneity in terms of sample size, treatment regimens, and outcome measures, and there is currently a lack of systematic evidence synthesis. Therefore, this study aims to conduct a systematic review of relevant RCTs from both domestic and international sources and employ meta-analysis methods to systematically evaluate the efficacy and safety of thymosin α1 combined with chemotherapy in the treatment of gastric cancer. The objective is to provide evidence-based medical support for clinical decision-making and to offer guidance for the design of future studies.

## 2 Methods

### 2.1 Study registration

This study was reported in strict accordance with the PRISMA guidelines (28) for reporting systematic reviews and meta-analyses (**Supplementary Material S1**). The study protocol has been registered with PROSPERO, under registration number CRD420261282679.

### 2.2 Search strategy

This study systematically searched following electronic databases: PubMed, Embase, the Cochrane Library, Web of Science, CNKI, Wanfang Data, SinoMed, and VIP. Search time frame spanned from inception of each database to January 14, 2026. Additionally, manual searches were conducted in the U.S. Clinical Trials Registry (https://clinicaltrials.gov/) and the China Clinical Trials Registry (https://www.chictr.org.cn/) to identify unpublished gray literature. Search strategy combined MeSH with free-text terms. Chinese search terms included: “胸腺肽α1,” “胸腺素α1,” “胃癌,” “胃恶性肿瘤,” and “化疗”; English search terms included: “thymosin alpha 1,” “thymalfasin,” “gastric cancer,” “stomach neoplasm,” and “chemotherapy.” Specific search strategies and results for each database are provided in **Supplementary Material S2**.

### 2.3 Inclusion and exclusion criteria

Criteria were established based on PICOS principles as follows:

Inclusion criteria include: (1) Participants: Patients with pathologically confirmed gastric cancer, regardless of race, gender, age, or disease stage; (2) Interventions: Treatment group receives thymosin α1 in combination with chemotherapy; route of administration, dosage, and duration of thymosin α1 are unrestricted; (3) Comparators: Control group receives chemotherapy alone; chemotherapy regimen is the same as that of treatment group; (4) Outcomes: Primary outcomes include ORR and overall survival (OS); secondary outcomes include DCR, progression-free survival (PFS), tumor marker levels, immune function indicators (e.g., CD3^+^%, CD4^+^%, CD8^+^%, CD4^+^/CD8^+^ ratio), and incidence of adverse events; (5) Study Design: RCTs, regardless of whether they were blinded; language is limited to Chinese and English.

Exclusion criteria include: (1) Non-RCTs, cohort studies, case-control studies, case reports, reviews, meta-analyses, and animal studies; (2) Studies in which treatment group used other immunomodulators or targeted drugs in combination with thymosin α1; (3) Studies in which control group did not receive single-agent chemotherapy or in which the chemotherapy regimen differed from that of treatment group; (4) Literature with incomplete data or from which valid data could not be extracted; (5) Duplicate publications.

### 2.4 Study selection and data extraction

2 researchers (Y.L. and X.S.) independently conducted literature screening and data extraction. First, duplicate records were removed using EndNote 21; then, studies that were clearly irrelevant were excluded by reviewing titles and abstracts; finally, full texts were read for a secondary screening. In case of disagreement, the issue was resolved through discussion or by a third researcher (R.Z.). The data extraction included: (1) Basic information; (2) Intervention details; (3) Outcome measures; (4) Study design. For studies with incomplete data, the corresponding authors were contacted via email to obtain raw data.

### 2.5 Risk of bias assessment

ROB 2 was used to assess risk of bias in included studies (29). The tool covers the following 5 domains: (1) bias arising from randomization process; (2) bias arising from deviation from assigned intervention; (3) bias arising from missing outcome data; (4) bias arising from outcome measurement; (5) bias due to selective reporting of results. Assessment results for each domain were categorized as “low risk,” “some concerns,” or “high risk,” and an overall risk of bias rating was derived from combined results. Assessment was conducted independently by 2 researchers (T.S. and H.Q.); in cases of disagreement, issue was resolved through discussion or adjudication by a third researcher (Z.W.).

### 2.6 Statistical analysis

Meta-analysis was conducted using Stata 18.0. For dichotomous variables, risk ratio (RR) and its 95% confidence interval (CI) were calculated; for continuous variables, weighted mean difference (WMD) was used if the measurement tools and units were consistent, and standardized mean difference (SMD) was used if they were inconsistent. Heterogeneity among included studies was assessed using I² statistic and Cochran Q test. If I² < 50% and P > 0.10, indicating low heterogeneity, a fixed-effects model was used; if I² ≥ 50% and P ≤ 0.10, indicating significant heterogeneity, a random-effects model was used (30).

For key outcomes with high heterogeneity, meta-regression analysis was first performed. The pre-specified covariates included: (1) patient age (elderly vs. non-elderly patients); (2) Thymosin α1 administration frequency (once daily vs. twice weekly vs. every other day); (3) Total Thymosin α1 treatment dose (stratified into low, medium, and high-dose groups based on tertiles). Meta-regression analysis was performed using Restricted Maximum Likelihood (REML) method; a P-value < 0.05 indicated that covariate might have a significant effect on effect size (31).

Subgroup analyses were conducted based on results of meta-regression. For covariates that did not show a significant effect in meta-regression, pre-specified subgroup analyses were still conducted based on clinical relevance as exploratory analyses. Sensitivity analyses were performed using stepwise exclusion method to assess the impact of individual studies on pooled effect size. Publication bias was qualitatively assessed using funnel plots and quantitatively assessed using Egger’s test (32).

### 2.7 Evaluation of evidence quality

GRADE system was used to grade quality of evidence for outcome measures (https://www.gradepro.org/) (33). Grading factors included risk of bias, inconsistency, indirectness, imprecision, and publication bias. Evidence quality was categorized into 4 levels: high, moderate, low, and very low. GRADE assessment results were presented in form of an evidence quality summary table.

### 2.8 Trial sequential analysis

To prevent random errors (Type I errors) resulting from repeated testing in cumulative meta-analyses, we performed TSA on selected outcome measures using TSA software v.0.9.5.10 Beta (Copenhagen Trial Unit, Copenhagen, Denmark). We pre-specified a Type I error rate (α) of 5% (two-sided) and a power (1-β) of 80%, and calculated required information volume based on existing study data. By constructing a TSA plot, we determined whether current cumulative evidence had crossed required null/significance boundary or whether further studies were still needed (34, 35).

## 3 Results

### 3.1 Characteristics of included studies

#### 3.1.1 Literature screening process

Initial search yielded a total of 565 articles; after deduplication using EndNote, 368 articles remained. After reviewing titles and abstracts, 254 clearly irrelevant articles were excluded, leaving 114 articles for full-text screening. Following full-text review, 93 articles were excluded for following reasons: reviews (5), animal or cell experiments (9), case reports (5), interventions that did not meet inclusion criteria (33), study populations that did not meet inclusion criteria (21), and non-RCTs (21). Ultimately, 20 RCTs (24–27, 36–52) were included for meta-analysis. Literature screening process is shown in **Figure 1**.

**Figure 1:**
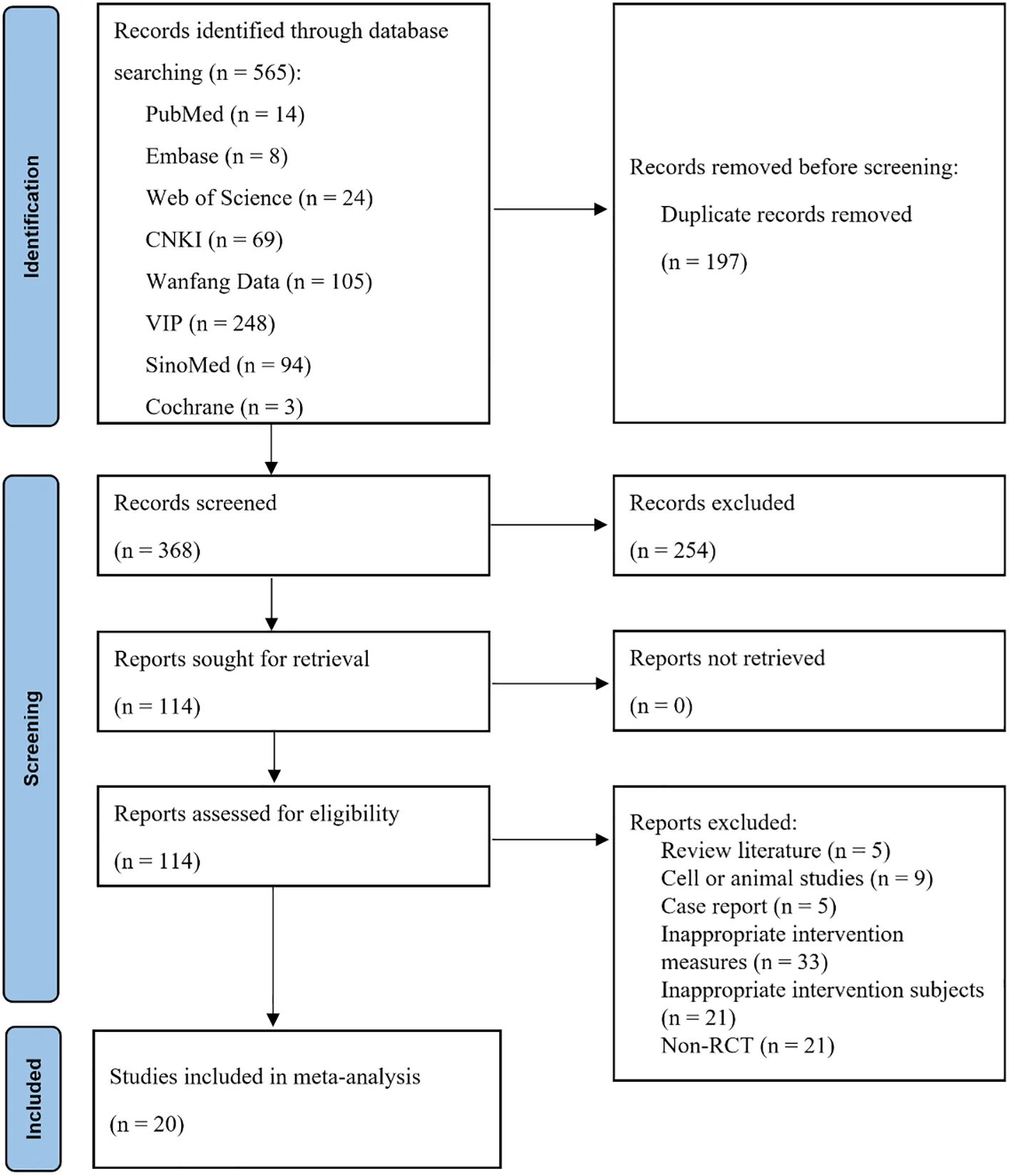
Flow diagram of the literature search.

#### 3.1.2 Characteristics of included studies

20 RCTs were included, published between 2008 and 2024, all of which were conducted in China. Total sample size was 1,704 gastric cancer patients, including 854 in treatment group and 850 in control group. Sample size of each study ranged from 56 to 150 patients.

Regarding interventions, treatment groups all received thymosin α1 in combination with chemotherapy. Thymosin α1 dosing regimens included: once daily (9 RCTs), twice weekly (9 RCTs), and every other day (2 RCTs); The single dose was 1.6 mg; treatment duration ranged from 3 to 24 weeks. Chemotherapy regimens included: XELOX (3 RCTs), SOX (4 RCTs), FOLFOX (7 RCTs), FOLFIRI (2 RCTs), and others (4 RCTs). Control groups all received the same chemotherapy regimens as treatment groups. The baseline characteristics of included studies are detailed in **Table 1**.

**Table 1:** The characteristics of all included studies.

Author and reference	Sample size		Mean age (years)		Interventions		Thymosin α1 administration method and total dose (injections)	Chemotherapy cycles (number)	Non-elderly (0) / Elderly (1)	Outcomes
	T	C	T	C	T	C				
Hou et al. (2008) (36)	28	28	53.2±13.1		Thymosin α1	FOLFOX4	Once daily (21)	1	0	③④
Lin et al. (2010) (37)	30	30	M57	M56	Thymosin α1	DCF	Twice weekly (12)	2	0	①②
Han et al. (2011) (38)	63	63	52.8(39-74)	52.1(40-73)	Thymosin α1	FOLFOX4	Once daily (56)	4	0	③④⑤
Wang et al. (2011) (39)	35	35	M52	M49	Thymosin α1	FP	Twice weekly (16)	2	0	③
Tian et al. (2015) (24)	45	45	55.34±4.82	53.62±4.27	Thymosin α1	FOLFOX4	Once daily (63)	3	0	①②③④⑨
Guo et al. (2016) (40)	40	40	68.93±9.28	69.65±10.04	Thymosin α1	FOLFOX4	Twice weekly (8)	2	1	①③⑦⑧
Shao et al. (2016) (41)	58	58	M55(42-70)		Thymosin α1	XELOX	Twice weekly (36-48)	3	0	①②③④⑤⑥
Xiao et al. (2016) (25)	28	26	M54(42-67)	M56(41-69)	Thymosin α1	SOX	Twice weekly (12)	2	0	①②⑨
Xu et al. (2016) (42)	38	38	80.2±7	76.2±5.3	Thymosin α1	SOX	Twice weekly (6)	1	1	①②⑤⑥
Liao et al. (2017) (43)	40	40	70.2±11.3		Thymosin α1	XELOX	Every other day (30)	3	1	①③
Liu et al. (2017) (44)	40	40	69.1±5.74		Thymosin α1	DOS	Every other day (31)	3	0	⑤
Mao et al. (2018) (45)	49	49	71.5±8.9	70.2±9.3	Thymosin α1	FOLFOX4	Once daily (63)	3	1	①②③④⑦⑧⑨
Zhang et al. (2018) (46)	45	45	71.16±7.72	72.93±7.75	Thymosin α1	FOLFOX6	Once daily (42)	3	1	①③
Guo et al. (2019) (47)	46	46	68.22±5.97	68.30±6.31	Thymosin α1	XELOX	Once daily (63)	3	1	①②③⑦⑨
Li et al. (2019) (26)	38	38	60.09±11.83	59.71±12.08	Thymosin α1	FOLFIRI	Once daily (42)	3	0	①②③⑤
Yan et al. (2019) (48)	31	31	54.94±4.61	54.12±4.38	Thymosin α1	FOLFIRI	Once daily (56)	4	0	①②③⑤
Zuo et al. (2019) (49)	29	29	59.14±4.33	60.27±4.07	Thymosin α1	DOS	Once daily (63)	3	0	①②⑤
Lin et al. (2020) (50)	45	43	M58(28-75)		Thymosin α1	SOX	Twice weekly (36-48)	6-8	0	①②③④⑤
Liu et al. (2021) (27)	75	75	57.35±7.11	56.57±6.94	Thymosin α1	SOX	Twice weekly (36)	6	0	①②③④⑨
Wang et al. (2024) (51)	51	51	67.72±2.24	67.21±2.24	Thymosin α1	FOLFOX4	Twice weekly (18)	3	1	①②③⑥⑦⑨

T, treatment group; C, control group.

Outcomes: ①ORR; ②DCR; ③T lymphocyte subsets (CD3^+^%, CD4^+^%, CD8^+^%, and CD4^+^/ CD8^+^); ④NK cell level; ⑤KPS improvement rate or KPS score; ⑥CEA or CA199; ⑦MMP-2 and MMP-9; ⑧TNF-α, IFN-γ, IL-4, and IL-10; ⑨Incidence of adverse drug reactions (ADRs).

### 3.2 Risk of bias assessment

Regarding the randomization process, 11 studies were rated as having “some concerns” because they only mentioned randomization without specifying the specific randomization method. Remaining 9 studies were rated as “low risk.” Regarding outcome measurement, 7 studies were rated as “high risk” because they involved subjective outcomes and did not mention use of blinding. All studies reported complete outcome data, and were rated as “low risk” for deviation from the intended intervention, missing outcome data, and selective reporting of results. See **Figure 2** for detailed results of risk of bias assessment.

**Figure 2:**
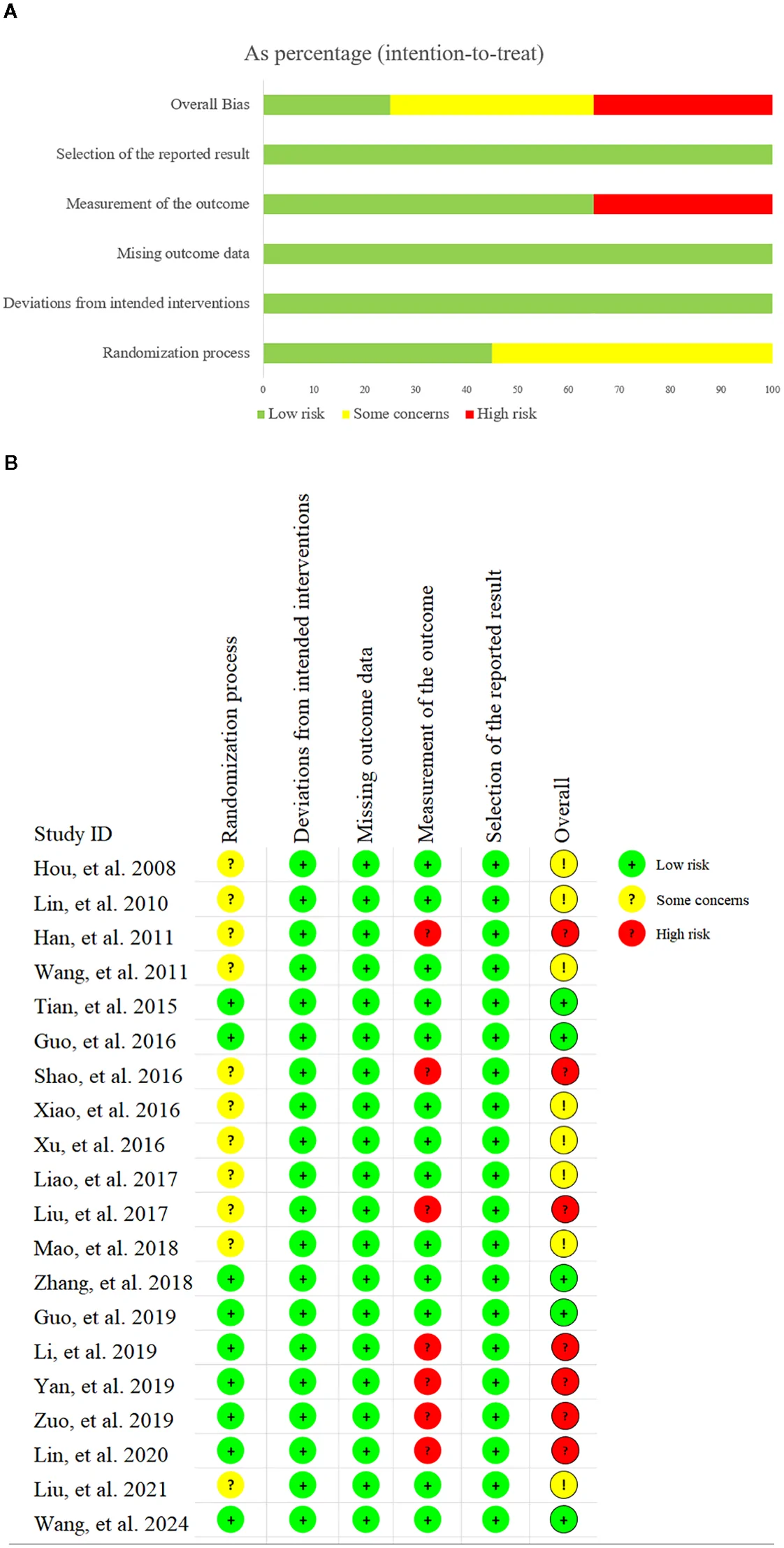
Risk of bias of included study. **(A)** Risk of bias summary. **(B)** Risk of bias graph.

### 3.3 Efficacy measures

#### 3.3.1 ORR

16 RCTs reported ORR. Meta-analysis results showed that ORR in thymosin α1 plus chemotherapy group was higher than that in chemotherapy-alone group (RR = 1.39, 95% CI 1.26–1.53, P < 0.00001). There was no heterogeneity among studies (I² = 0%, P = 0.935), and a fixed-effects model was used for analysis (**Figure 3**).

**Figure 3:**
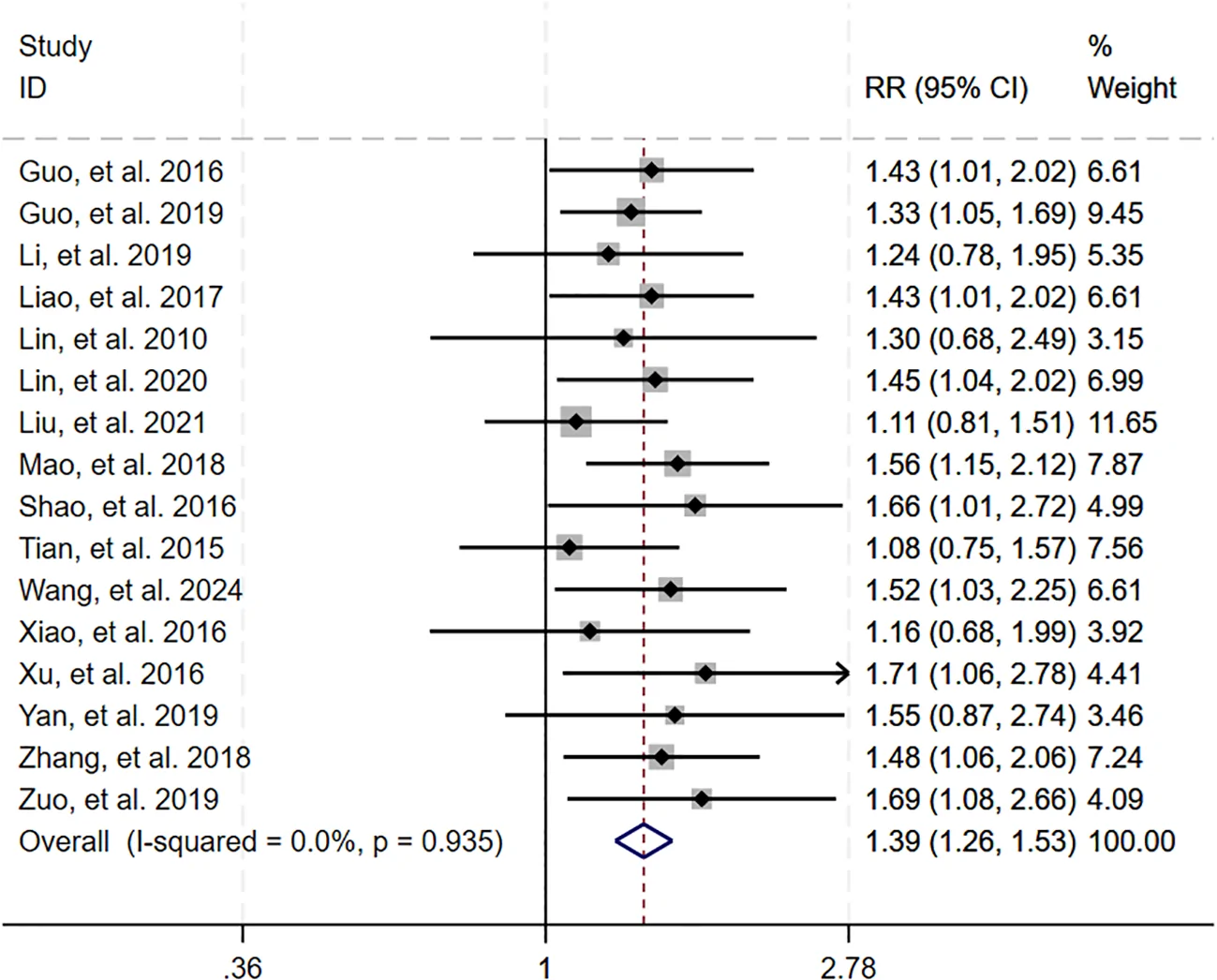
Forest plot of ORR.

#### 3.3.2 DCR

13 RCTs reported DCR. Meta-analysis results showed that DCR in thymosin α1 plus chemotherapy group was higher than that in chemotherapy-alone group (RR = 1.16, 95% CI 1.10–1.24, P < 0.00001). There was no heterogeneity among studies (I² = 5.8%, P = 0.388), and a fixed-effects model was used for analysis (**Figure 4**).

**Figure 4:**
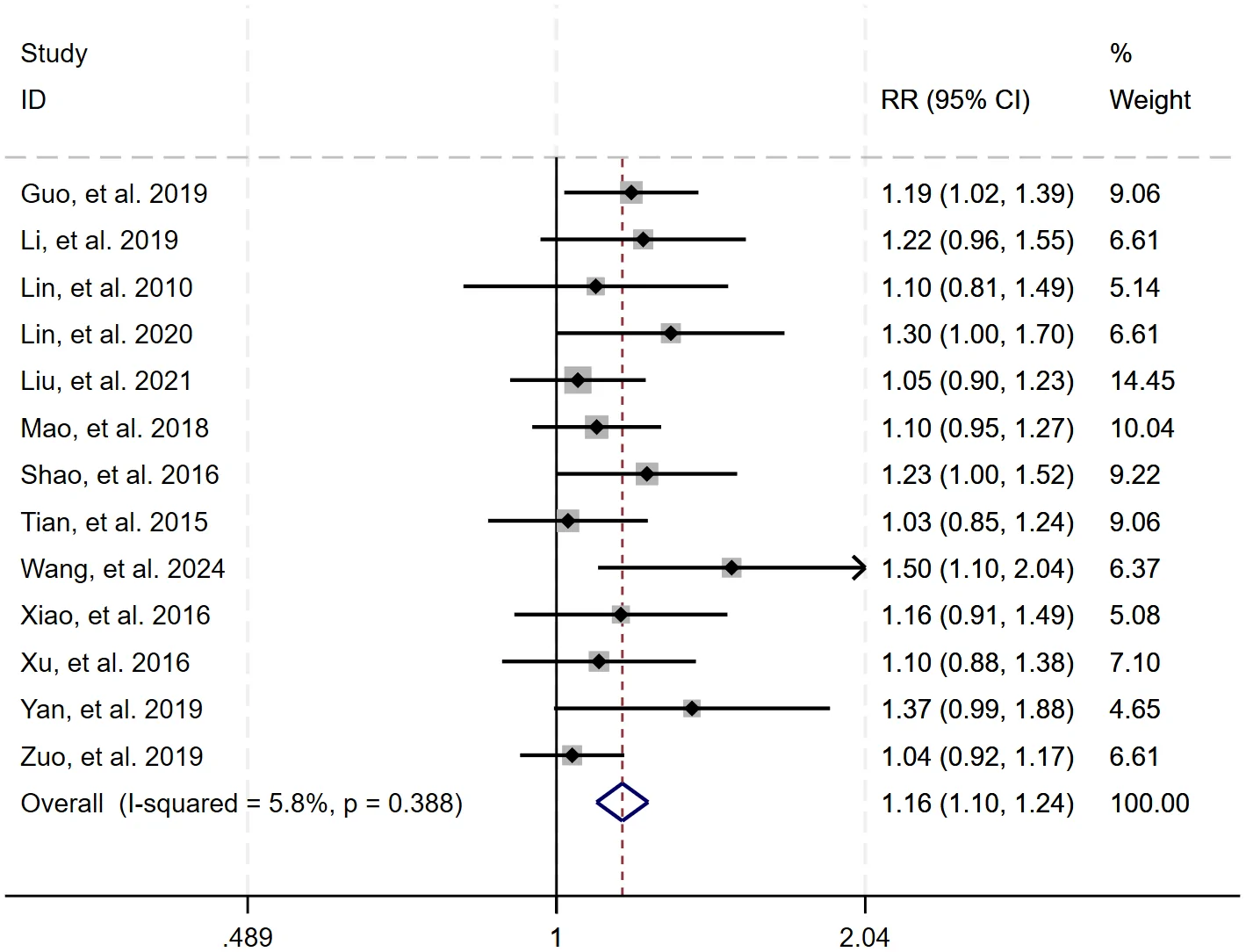
Forest plot of DCR.

#### 3.3.3 KPS score and KPS improvement rate

5 RCTs reported KPS scores. Results showed that KPS score in thymosin α1 plus chemotherapy group was higher than that in chemotherapy-alone group (WMD = 8.27, 95% CI 4.21–12.32, P < 0.0001). There was high heterogeneity among studies (I² = 93.4%, P = 0), and a random-effects model was used for analysis (**Figure 5A**).

**Figure 5:**
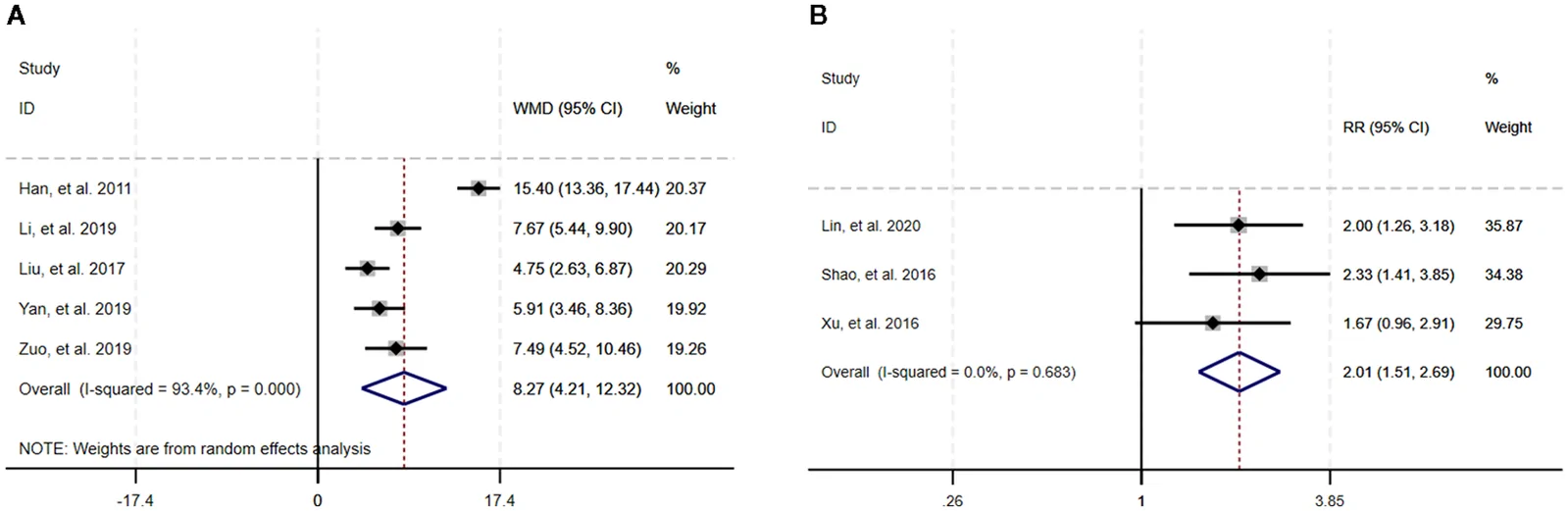
**(A)** Forest plot of KPS. **(B)** Forest plot of KPS improvement rate.

3 RCTs reported KPS improvement rate. Results showed that KPS improvement rate in thymosin α1 plus chemotherapy group was higher than that in chemotherapy-alone group (RR = 2.01, 95% CI 1.51–2.69, P < 0.00001). There was no heterogeneity among studies (I² = 0%, P = 0.683), and a fixed-effects model was used for analysis (**Figure 5B**).

### 3.4 Immunological parameters

#### 3.4.1 CD3^+^%

7 RCTs reported CD3^+^%. Results showed that CD3^+^% in thymosin α1 plus chemotherapy group was higher than that in chemotherapy-alone group (WMD = 7.73, 95% CI 5.60–9.87, P < 0.00001). There was high heterogeneity among studies (I² = 72.3%, P = 0.001), and a random-effects model was used for analysis (**Figure 6A**).

**Figure 6:**
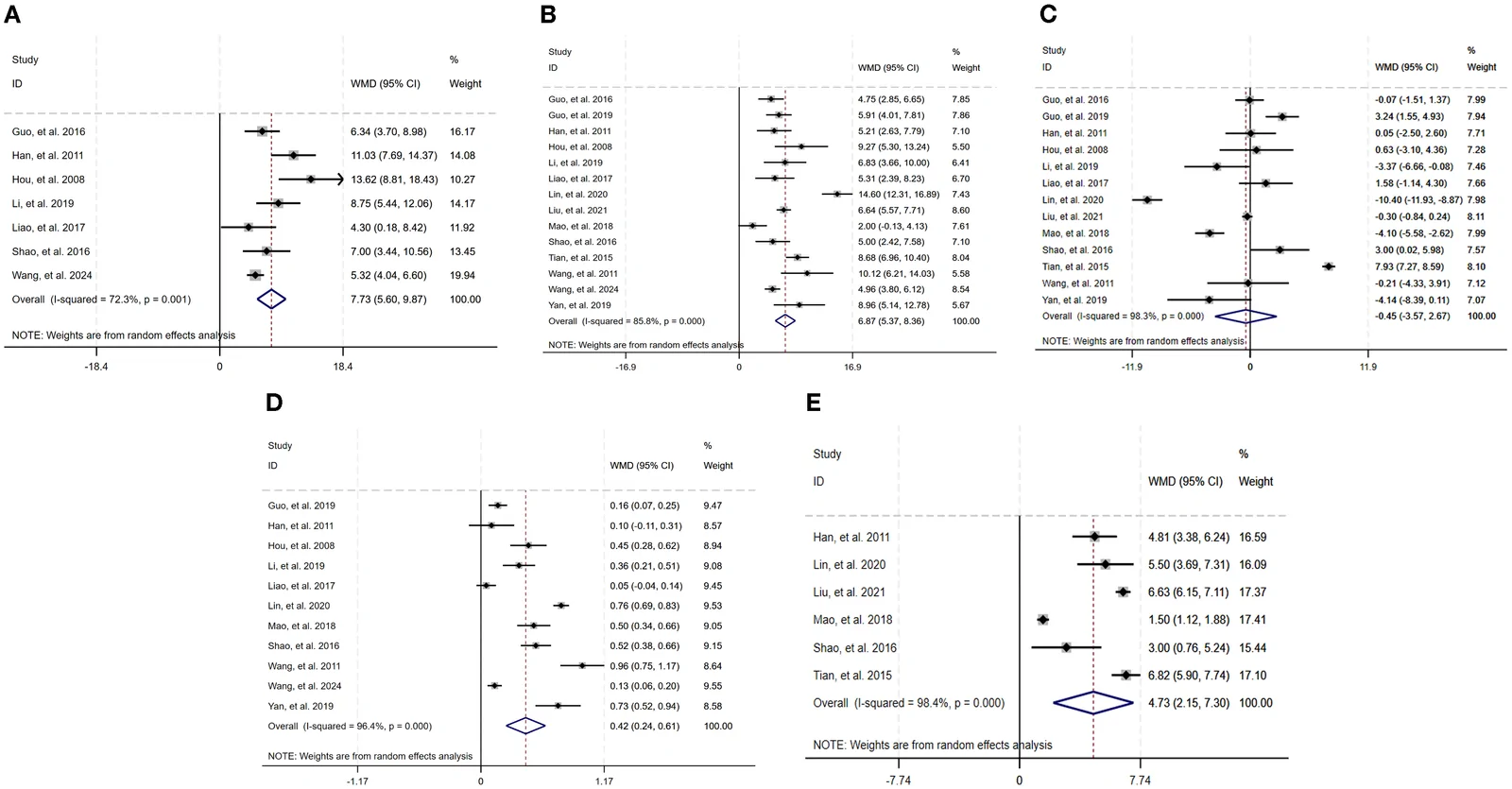
Forest plot of immunological parameters. **(A)** Forest plot of CD3^+^%. **(B)** Forest plot of CD4^+^%. **(C)** Forest plot of CD8^+^%. **(D)** Forest plot of CD4^+^/CD8^+^ ratio. **(E)** Forest plot of NK.

#### 3.4.2 CD4^+^%

14 RCTs reported CD4^+^%. Results showed that CD4^+^% in thymosin α1 plus chemotherapy group was higher than that in chemotherapy-alone group (WMD = 6.87, 95% CI 5.37–8.36, P < 0.0001). There was high heterogeneity among studies (I² = 85.8%, P = 0.0001), and a random-effects model was used (**Figure 6B**).

#### 3.4.3 CD8^+^%

13 RCTs reported CD8^+^%. Results showed no significant difference in CD8^+^% between 2 groups (WMD = −0.45, 95% CI −3.57 to 2.67, P = 0.78). There was high heterogeneity among studies (I² = 98.3%, P = 0), and a random-effects model was used (**Figure 6C**).

#### 3.4.4 CD4^+^/CD8^+^ ratio

11 RCTs reported CD4^+^/CD8^+^ ratio. Meta-analysis results showed that CD4^+^/CD8^+^ ratio in thymosin α1 plus chemotherapy group was higher than that in chemotherapy-alone group (WMD = 0.42, 95% CI 0.24–0.61, P < 0.0001). There was high heterogeneity among studies (I² = 96.4%, P = 0), and a random-effects model was used (**Figure 6D**).

#### 3.4.5 Natural killer cells

6 RCTs reported NK cell counts. Meta-analysis results showed that NK cell counts in thymosin α1 plus chemotherapy group were higher than those in chemotherapy-alone group (WMD = 4.73, 95% CI 2.15–7.30, P = 0.0003). There was high heterogeneity among studies (I² = 98.4%, P = 0), and a random-effects model was used (**Figure 6E**).

### 3.5 Tumor markers

#### 3.5.1 Carcinoembryonic antigen

3 RCTs reported serum CEA levels. Results showed that CEA level in thymosin α1 plus chemotherapy group was lower than those in chemotherapy-alone group (WMD = -5.48, 95% CI -10.58 to -0.39, P = 0.03). There was high heterogeneity among studies (I² = 81.5%, P = 0.005), and a random-effects model was used (**Figure 7A**).

**Figure 7:**
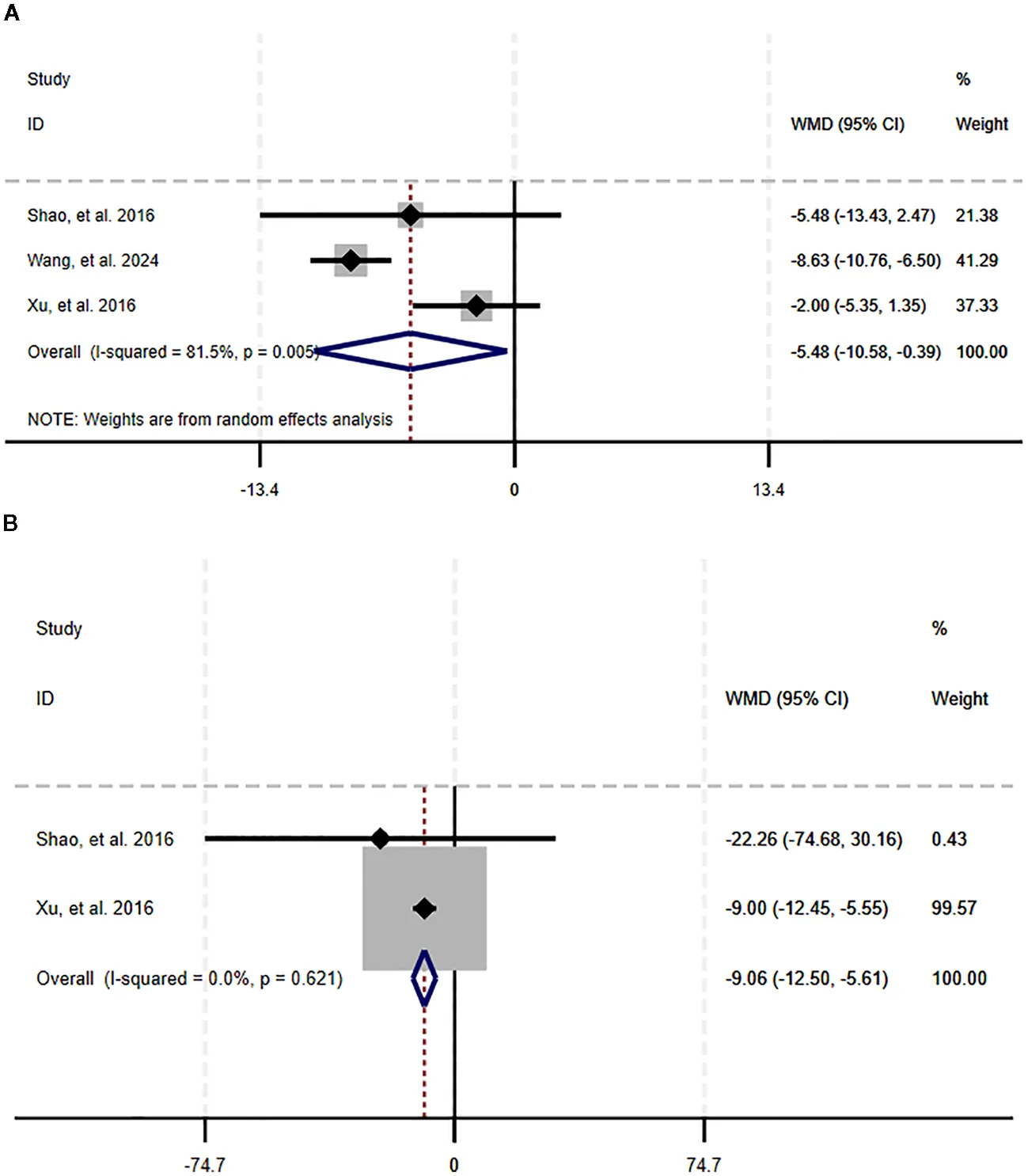
Forest plot of tumor markers. **(A)** Forest plot of CEA. **(B)** Forest plot of CA199.

#### 3.5.2 Carbohydrate antigen 19-9

2 RCTs reported serum CA19–9 levels. Results showed that CA19–9 level in thymosin α1 plus chemotherapy group was lower than those in chemotherapy-alone group (WMD = −9.06, 95% CI −12.50 to −5.61, P < 0.00001). There was no heterogeneity among studies (I² = 0%, P = 0.621), and a fixed-effects model was used (**Figure 7B**).

### 3.6 Inflammatory cytokines

4 RCTs reported changes in serum inflammatory cytokine levels before and after treatment. Results showed that thymosin α1 combined with chemotherapy demonstrated an advantage in modulating inflammatory cytokine profile. Combination therapy group exhibited reductions in matrix metalloproteinase-2 (MMP-2) (WMD = -25.60, 95% CI -43.56 to -7.64, P = 0.005; 4 RCTs), and matrix metalloproteinase-9 (MMP-9) (WMD = -50.63, 95% CI -62.98 to -38.27, P < 0.00001; 4 RCTs) were lower than those in chemotherapy-alone group. Levels of interleukin-4 (IL-4) (WMD = -2.12, 95% CI -2.41 to -1.82, P < 0.00001; 2 RCTs) and interleukin-10 (IL-10) (WMD = -1.01, 95% CI -1.82 to -0.21, P = 0.01; 2 RCTs) were also lower than those in control group. In addition, levels of interferon-γ (IFN-γ) (WMD = 1.67, 95% CI 0.94–2.41, P < 0.00001; 2 RCTs) and tumor necrosis factor-α (TNF-α) (WMD = 3.33, 95% CI 2.19–4.47, P < 0.00001; 2 RCTs) were higher in combination therapy group than in chemotherapy-alone group. Specific results are shown in **Figure 8**.

**Figure 8:**
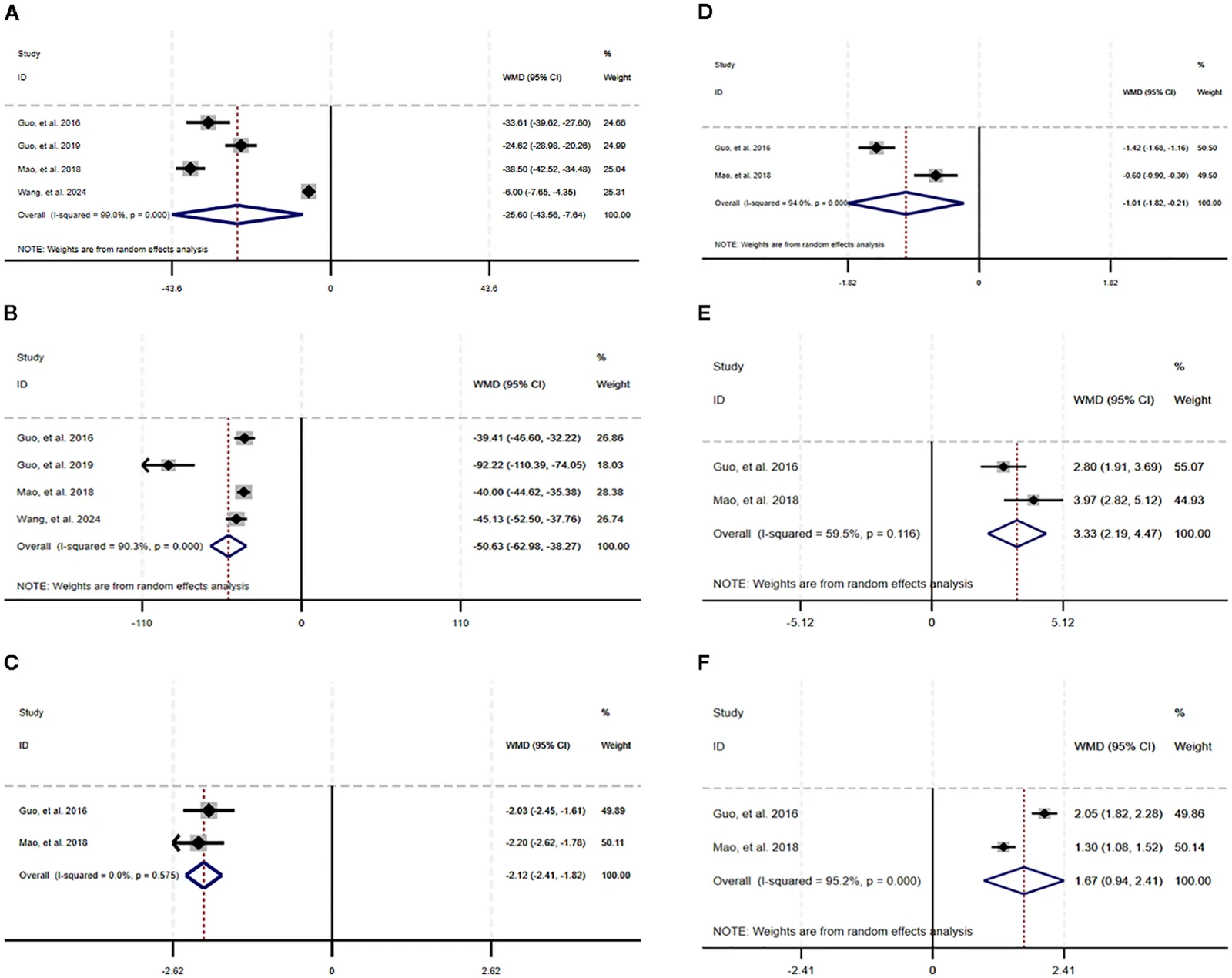
Forest plot of inflammatory cytokines. **(A)** Forest plot of MMP-2. **(B)** Forest plot of MMP-9. **(C)** Forest plot of IL-4. **(D)** Forest plot of IL-10. **(E)** Forest plot of IFN-γ. **(F)** Forest plot of TNF-α.

### 3.7 Incidence of adverse reactions

#### 3.7.1 Gastrointestinal reactions

7 RCTs reported incidence of gastrointestinal reactions. Results showed that incidence of gastrointestinal reactions in thymosin α1 plus chemotherapy group was lower than that in chemotherapy-alone group (RR = 0.63, 95% CI 0.44–0.89, P = 0.01). There was mild heterogeneity among studies (I² = 64.8%, P = 0.009), and a random-effects model was used (**Figure 9A**).

**Figure 9:**
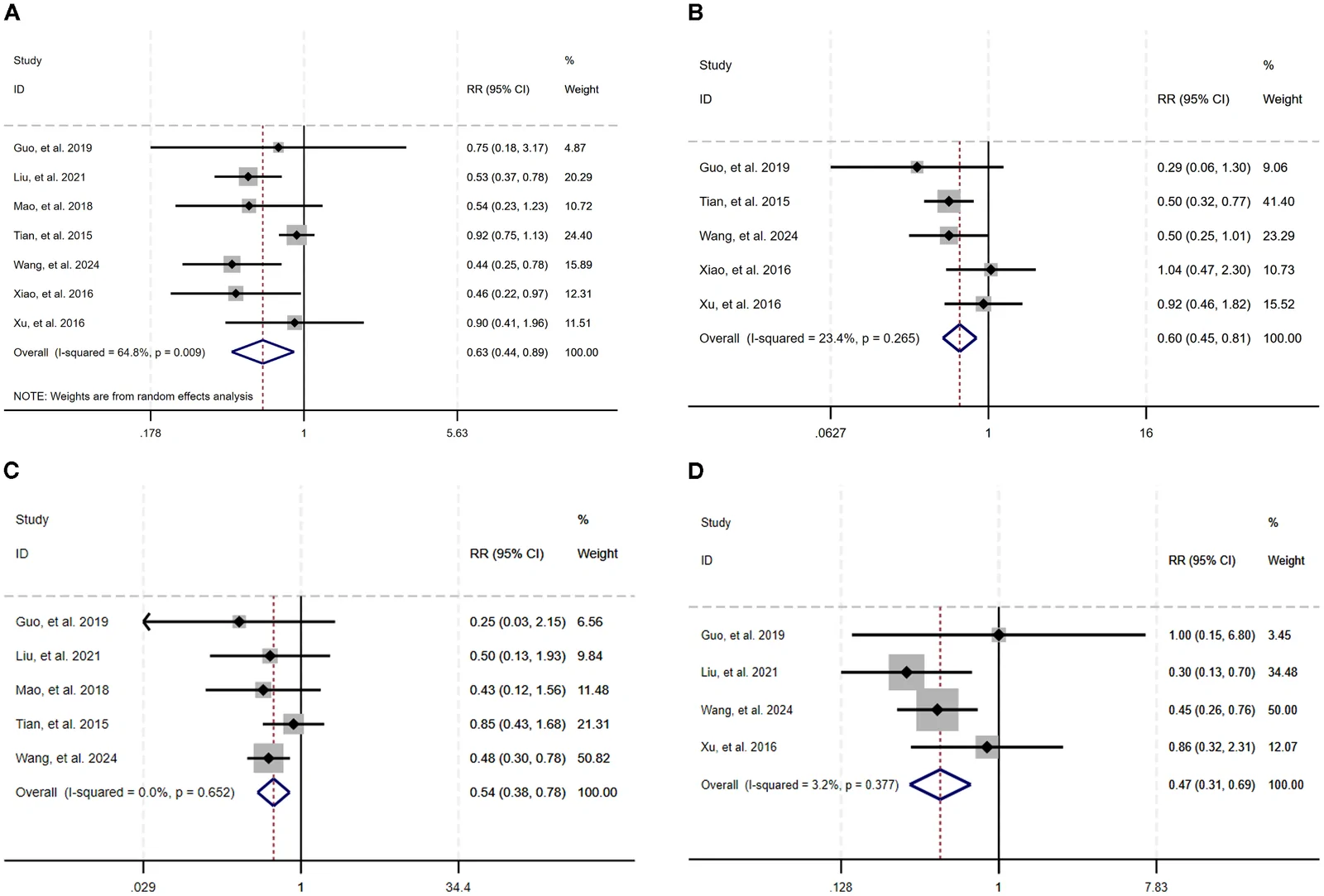
Forest plot of incidence of adverse reactions. **(A)** Forest plot of gastrointestinal reactions. **(B)** Forest plot of myelosuppression. **(C)** Forest plot of abnormal liver function. **(D)** Forest plot of neurotoxicity.

#### 3.7.2 Myelosuppression

5 RCTs reported incidence of myelosuppression. Results showed that incidence of myelosuppression in thymosin α1 plus chemotherapy group was lower than that in chemotherapy-alone group (RR = 0.60, 95% CI 0.45–0.81, P = 0.001). There was mild heterogeneity among studies (I² = 23.4%, P = 0.265), and a fixed-effects model was used (**Figure 9B**>).

#### 3.7.3 Abnormal liver function

5 RCTs reported incidence of abnormal liver function. Meta-analysis results showed that incidence of liver function abnormalities in thymosin α1 plus chemotherapy group was lower than in chemotherapy-alone group (RR = 0.54, 95% CI 0.38–0.78, P = 0.0008). There was no heterogeneity among studies (I² = 0%, P = 0.652), and a fixed-effects model was used (**Figure 9C**).

#### 3.7.4 Neurotoxicity

4 RCTs reported incidence of neurotoxicity. Results showed that incidence of neurotoxicity in thymosin α1 plus chemotherapy group was lower than that in chemotherapy-alone group (RR = 0.47, 95% CI 0.31–0.69, P = 0.0002). There was no heterogeneity among studies (I² = 3.2%, P = 0.377), and a fixed-effects model was used (**Figure 9D**).

### 3.8 Results of meta-regression analysis

A meta-regression analysis was conducted to investigate potential effects of modifying factors on selected outcomes with a large number of included studies (ORR, DCR, CD3^+^%, CD4^+^%, CD8^+^%, CD4^+^/CD8^+^ ratio, NK, and gastrointestinal reactions). Patient age, frequency of thymosin α1 administration, and total dose of thymosin α1 were included as covariates. Results showed that none of 3 covariates had a significant modifying effect on all outcomes. Specific results of meta-regression analysis are presented in **Table 2**.

**Table 2:** Results of meta-regression analysis.

Outcomes	Patient age	Thymosin α1 administration frequency	Total Thymosin α1 treatment dose
ORR	P = 0.168	P = 0.722	P = 0.660
DCR	P = 0.355	P = 0.292	P = 0.526
CD3^+^%	P = 0.172	P = 0.323	P = 0.328
CD4^+^%	P = 0.139	P = 0.608	P = 0.981
CD8^+^%	P = 0.865	P = 0.703	P = 0.671
CD4^+^/CD8^+^ ratio	P = 0.116	P = 0.629	P = 0.967
NK	P = 0.125	P = 0.436	P = 0.337
Gastrointestinal reactions	P = 0.713	P = 0.384	P = 0.794

### 3.9 Results of subgroup analysis

Although the meta-regression analysis did not reveal any significant moderating effects of 3 covariates on any of outcomes, we nevertheless conducted operational exploratory subgroup analyses for some of outcomes (ORR, DCR, CD3+%, CD4+%, CD4^+^/CD8^+^ ratio) in an effort to identify trends in the varying efficacy of thymosin α1 for gastric cancer patients across different clinical settings. The following is a brief summary of our subgroup analyses for ORR and CD4^+^/CD8^+^ ratio; for subgroup analyses of other outcome measures, please refer to **Supplementary Material S3**.

#### 3.9.1 Subgroup analysis of ORR

Stratification by patient age: In elderly patient group (7 RCTs), ORR for thymosin α1 combined with chemotherapy was superior to that of chemotherapy alone (RR = 1.48, 95% CI 1.30–1.68); In non-elderly patient group (9 RCTs), a benefit was also observed (RR = 1.31, 95% CI 1.13–1.51).

Stratification by dosing frequency: In twice-weekly group (8 RCTs), ORR was higher (RR = 1.39, 95% CI 1.20–1.60); In once-daily group (7 RCTs), an increase was also observed (RR = 1.39, 95% CI 1.21–1.59).

By total dose: In low-dose group (5 RCTs), ORR was higher (RR = 1.45, 95% CI 1.18–1.77); In moderate-dose group (2 RCTs), no significant difference was observed between 2 groups (RR = 1.22, 95% CI 0.97–1.54). In high-dose group (9 RCTs), an increase was also observed (RR = 1.42, 95% CI 1.26–1.61).

#### 3.9.2 Subgroup analysis of CD4^+^/CD8^+^ ratio

Stratified by patient age: In elderly patient group (4 RCTs), CD4^+^/CD8^+^ ratio was higher with thymosin α1 combined with chemotherapy than with chemotherapy alone (WMD = 0.19, 95% CI 0.06–0.33); In non-elderly patient group (7 RCTs), a benefit was also observed (WMD = 0.56, 95% CI 0.37–0.74).

Stratification by dosing frequency: In twice-weekly group (4 RCTs), CD4^+^/CD8^+^ ratio increased (WMD = 0.46, 95% CI 0.11–0.82); in once-daily group (6 RCTs), an increase was also observed (WMD = 0.46, 95% CI 0.22–0.71).

Stratification by total dose: In low-dose group (2 RCTs) and in medium-dose group (2 RCTs), there was no significant difference between 2 groups (WMD = 0.28, 95% CI -0.03 to 0.59; WMD = 0.50, 95% CI −0.39 to 1.39). In high-dose group (7 RCTs), an increase was also observed (WMD = 0.45, 95% CI 0.23–0.67).

### 3.10 Sensitivity analysis

Results showed that after sequentially excluding each study, there was no directional change in pooled effect sizes for primary outcomes such as ORR, DCR, KPS scores, and immune function indicators, indicating that Meta-analysis results are robust. Only for abnormal liver function and myelosuppression did the pooled effect lose statistical significance after excluding a single study (**Figures 10D, E**). Main results of sensitivity analysis (ORR, CD4^+^/CD8^+^, and gastrointestinal reactions) are shown in **Figure 10**; complete results are available in **Supplementary Material S3**.

**Figure 10:**
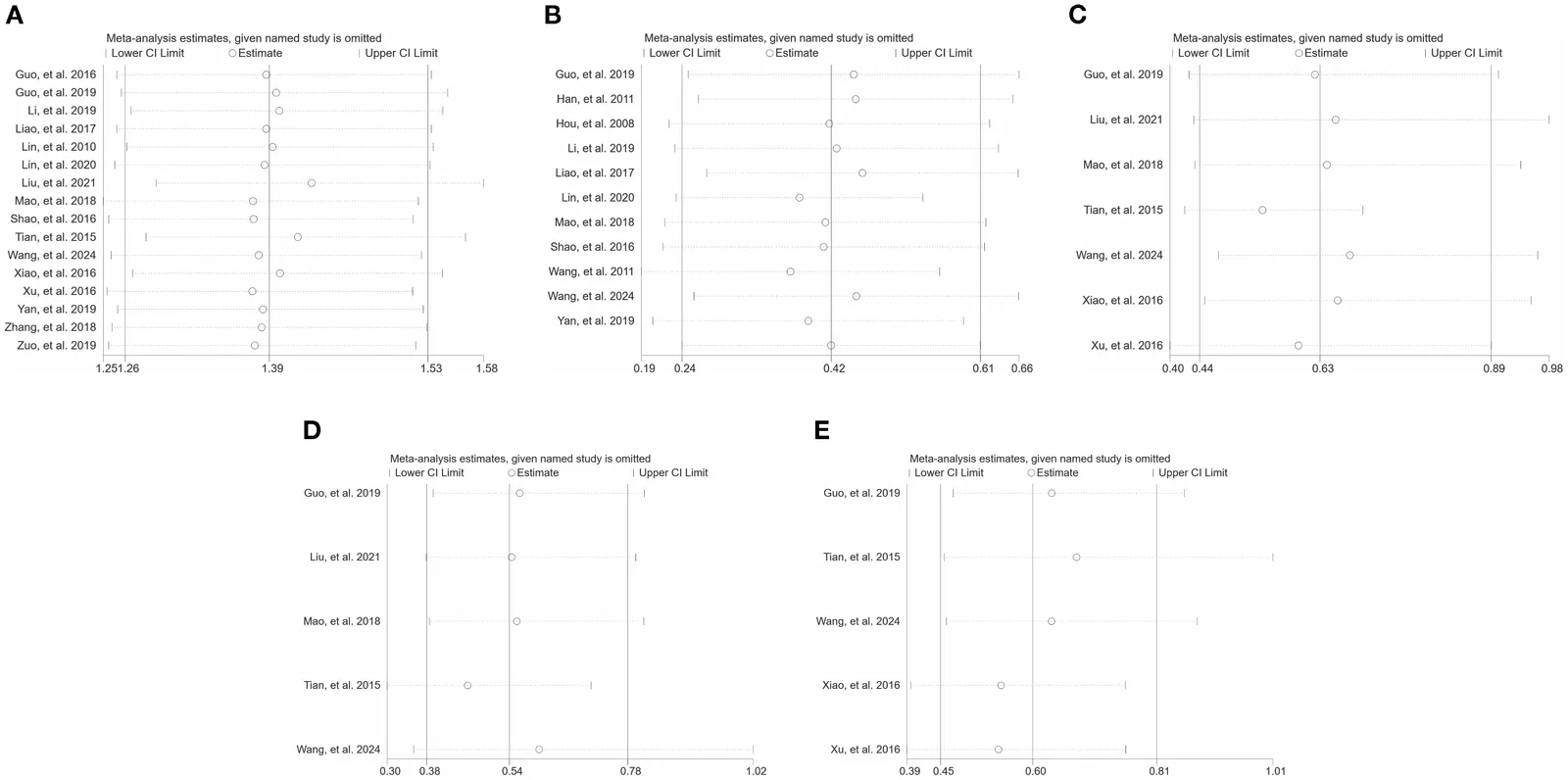
Sensitivity analyses of primary outcomes. **(A)** Sensitivity analyses of ORR. **(B)** Sensitivity analyses of CD4^+^/CD8^+^ ratio. **(C)** Sensitivity analyses of gastrointestinal reactions. **(D)** Sensitivity analyses of abnormal liver function. **(E)** Sensitivity analyses of myelosuppression.

### 3.11 Assessment of publication bias

For outcome measures with ≥10 included studies, publication bias was assessed using funnel plots combined with Egger’s test. Results showed that ORR (P = 0.5998), CD4^+^% (P = 0.2527), CD8^+^% (P = 0.6128), and CD4^+^/CD8^+^ ratio (P = 0.2020) all indicated no significant publication bias. Significant publication bias was observed for DCR (P = 0.0161); therefore, the “trim-and-fill” method was further applied. Results indicated that impact of publication bias on outcome was minimal (Observed: RR = 1.127, 95% CI 1.068–1.189; Observed + Imputed: RR = 1.084, 95% CI 1.033–1.138). Funnel plot results are shown in **Figure 11** and **Supplementary Material S3**.

**Figure 11:**
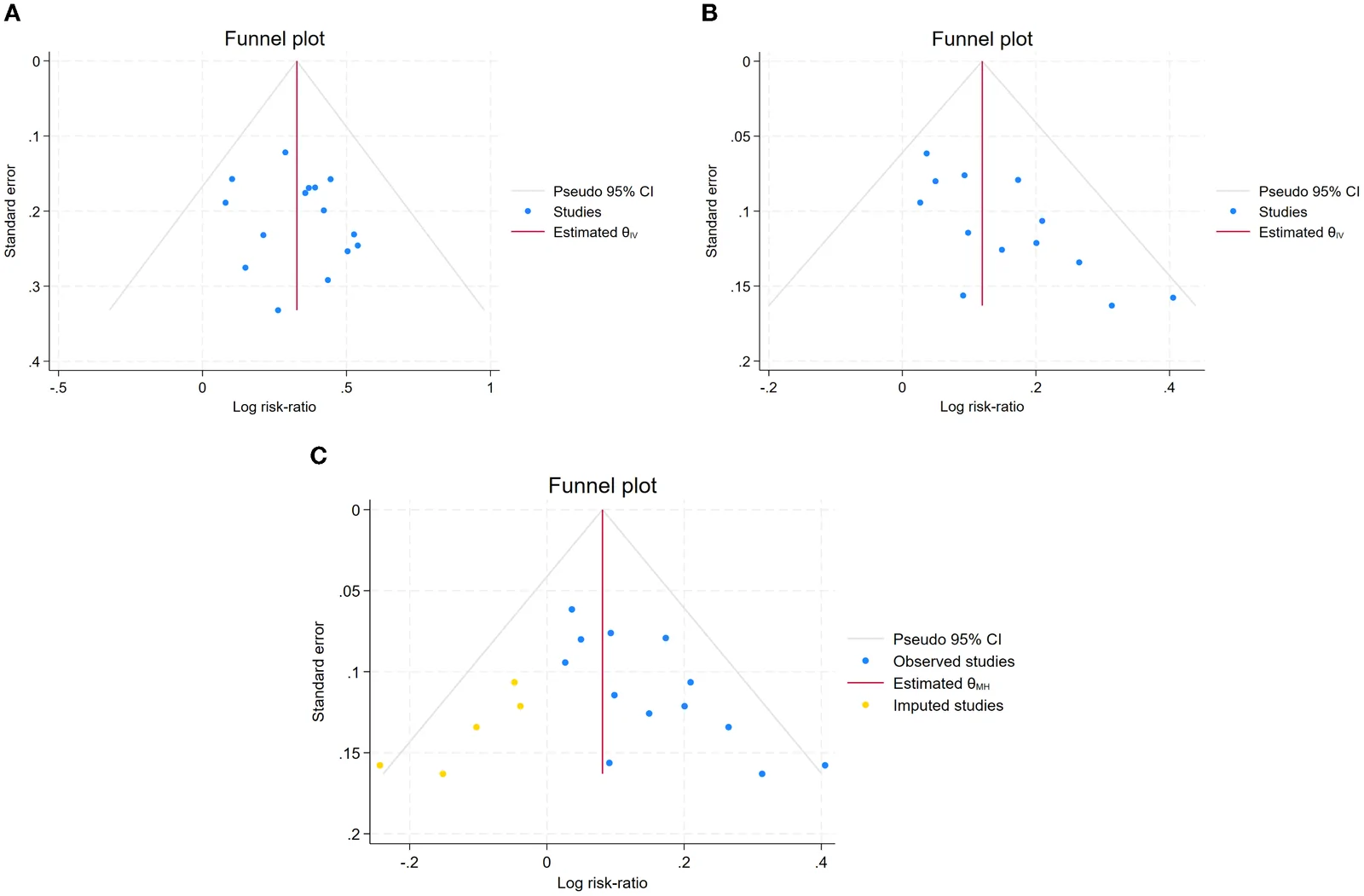
Funnel plot of primary outcomes. **(A)** Funnel plot of ORR. **(B)** Funnel plot of DCR. **(C)** After “trim and fill” method of funnel plot of DCR.

### 3.12 GRADE assessment of evidence quality

Quality of evidence for ORR was moderate; for DCR, it was low; for improvement in KPS score, it was low; and for KPS score, it was very low. The quality of evidence for immune function markers, tumor markers, and inflammatory factors ranged from very low to low; and for incidence of adverse reactions, it ranged from very low to low. GRADE evidence quality assessment results and reasons for downgrading are shown in **Supplementary Material S4**.

### 3.13 TSA

TSA results showed that, for outcomes such as ORR (RRR = 20%), DCR (RRR = 10%), CD4/CD8 ratio, myelosuppression, and NK cell levels, the cumulative Z-curves crossed traditional and trial-specified sequential monitoring thresholds. Specifically, ORR (RRR = 20%) and CD4/CD8 ratio have reached sample size required to draw conclusions. However, sample sizes for DCR (RRR = 10%), myelosuppression, and NK cell levels have not yet reached the required level of statistical power. Furthermore, for gastrointestinal reactions, although the cumulative Z-curve crossed the conventional threshold, it did not cross the trial’s sequential monitoring threshold. Results are shown in **Figure 12** and **Supplementary Material S3**.

**Figure 12:**
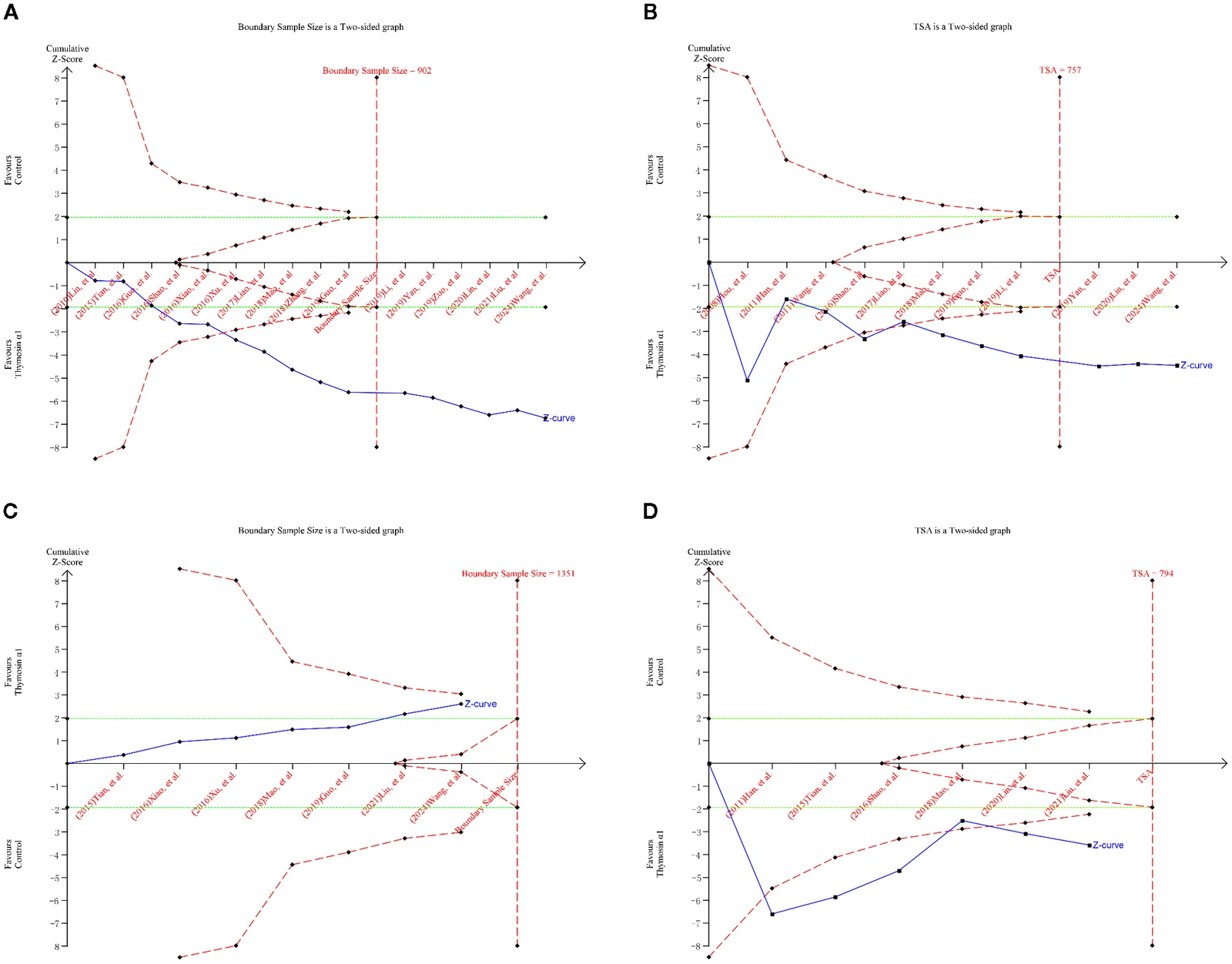
TSA results of primary outcomes. **(A)** TSA result of ORR (RRR=20%). **(B)** TSA result of CD4^+^/CD8^+^ ratio. **(C)** TSA result of gastrointestinal reactions. **(D)** TSA result of NK.

However, it is worth noting that TSA and GRADE have different focuses in evidence assessment and are complementary to one another—TSA is primarily used to control the risk of random errors (i.e., Type I and Type II errors), while GRADE comprehensively evaluates the overall certainty of evidence. The two should be interpreted in conjunction to comprehensively assess reliability of conclusions. With regard to this study, TSA results indicate that cumulative sample size has reached the information required to detect prespecified effect size, suggesting that current conclusions are statistically consistent and that there is no significant risk of random error. However, this result does not mitigate risk of bias arising from low quality of individual studies included in the analysis, nor does it alter the overall assessment of low certainty of evidence indicated by GRADE evaluation. Therefore, more precise and reliable conclusions still depend on future validation through additional high-quality, rigorously designed, and standardized RCTs.

## 4 Discussion

### 4.1 Summary of key findings

This study represents the first systematic evaluation of efficacy and safety of thymosin α1 combined with chemotherapy in the treatment of gastric cancer. Meta-analysis results indicate that combination of thymosin α1 and chemotherapy may not only enhance the efficacy of chemotherapy and improve patients’ quality of life, but may also boost patients’ immune function. Additionally, Thymosin α1 may modulate multiple cytokines. Regarding safety, combination therapy was associated with a reduced incidence of adverse reactions such as gastrointestinal reactions, myelosuppression, abnormal liver function, and neurotoxicity. These results suggest that combination of thymosin α1 and chemotherapy for gastric cancer has the potential to improve short-term efficacy, immune function, and quality of life, as well as enhance tolerability; however, due to the lack of long-term survival data, the long-term benefits for gastric cancer patients remain unclear.

To further clarify appropriate patient population for thymosin α1 combined with chemotherapy and to optimize dosing regimen, this study conducted a subgroup analysis based on patient age, dosing frequency, and total dose. The results showed no statistically significant differences in efficacy among the subgroups, consistent with the findings of meta-regression analysis. This suggests that the efficacy of thymosin α1 in combination with chemotherapy may be relatively stable and is not significantly influenced by patient age, dosing frequency, or total dose. This finding suggests that this combination regimen may have broad applicability, and its use in clinical practice may not need to be restricted by the aforementioned factors. However, it should be noted that the number of studies in some subgroups was very limited, which may have restricted the statistical power of the meta-regression analysis; therefore, these results should be interpreted with caution, and further high-quality studies are needed to clarify the dose-response relationship.

### 4.2 Discussion on the mechanism of action of thymosin α1

Thymosin α1 is an immunologically active peptide composed of 28 amino acids; its immunomodulatory mechanism is complex and involves multiple targets. Based on the results of this study and existing literature, the combined effect of thymosin α1 and chemotherapy may be related to the following mechanisms:

#### 4.2.1 Regulation of immune cell subpopulations

In this study, the increase in CD3^+^% and CD4^+^%, as well as improvement in CD4^+^/CD8^+^ ratio, in combination therapy group suggest that thymosin α1 may effectively reversed the immunosuppressive state induced by chemotherapy. Basic research indicates that thymosin α1 promotes dendritic cell maturation and antigen-presenting function via the Toll-like receptor (TLR) signaling pathway, thereby activating T-cell-mediated adaptive immune responses (52, 53). Notably, this study observed no significant difference in CD8^+^% between 2 groups, which may be related to higher sensitivity of CD8^+^ T cells to chemotherapy drugs or to relatively weaker regulatory effect of thymosin α1 on CD8^+^ T cells (54, 55). Studies have shown that thymosin α1 exerts a more significant transcriptional regulatory effect on activated CD8^+^ T cells, while its impact on resting CD8^+^ T cells is relatively minor (55). The increase in NK cell counts further supports the potential activating effect of thymosin α1 on the innate immune system, which is consistent with previous reports that thymosin α1 promotes the reactivation of NK cells (56, 57).

#### 4.2.2 Redistribution of the Th1/Th2 immune balance

The state of tumor immune microenvironment determines the efficacy of antitumor immune response. In this study, levels of IFN-γ and TNF-α (Th1 cytokines) were elevated in combination therapy group, while levels of IL-4 and IL-10 (Th2 cytokines) were reduced, suggesting that thymosin α1 combined with chemotherapy may promote a trend toward a shift in the antitumor immune response from Th2-dominant to Th1-dominant. Th1-type immune response is centered on cellular immunity and exerts direct antitumor effects by activating cytotoxic T lymphocytes and natural killer cells; in contrast, Th2-type immune response is associated with humoral immunity, and when overactivated, it can suppress Th1-type response and promote tumor immune evasion. As early as the 1990s, Garaci and colleagues proposed that thymosin α1 promotes production of Th1 cytokines and suppresses Th2 immune response (58). The changes in biomarkers observed in this study at the clinical level may reflect this mechanism, suggesting that restoration of Th1/Th2 balance may be one of the key mechanisms underlying the synergistic antitumor effects of thymosin α1 and chemotherapy, warranting further in-depth investigation in the future (59).

#### 4.2.3 Inhibition of factors associated with tumor invasion and metastasis

Furthermore, the regulation of tumor immune microenvironment involves not only T-cell subsets but is also closely related to polarization state of tumor-associated macrophages. M1-type macrophages possess antitumor activity, secreting pro-inflammatory factors such as IL-12 and TNF-α to activate Th1-type immune responses; whereas M2 macrophages promote tumor progression by secreting anti-inflammatory factors such as IL-4 and IL-10, and highly expressing matrix metalloproteinases (MMP-2 and MMP-9), which facilitate tumor invasion and metastasis by degrading the basement membrane and extracellular matrix (60). In this study, the significant reduction in M2-associated factors (IL-4, IL-10) and matrix metalloproteinases (MMP-2, MMP-9) in combination therapy group, accompanied by an increase in M1-associated factors (TNF-α), suggests that thymosin α1 may exert its effects by regulating polarization state of macrophages. A recent study by Liu et al. provides direct evidence for this mechanism, demonstrating that thymosin α1 can reverse M2 polarization of macrophages induced by oncogenic adenovirus, promoting their conversion to M1 phenotype, thereby enhancing antitumor immunity and therapeutic efficacy (61).

In summary, thymosin α1 may improve the tumor immune microenvironment through multifaceted mechanisms, thereby potentially inhibiting tumor invasion and metastasis. However, the results of meta-analysis do not directly support this proposed mechanism, and evidence from basic research remains relatively scarce. Further mechanism-oriented studies are needed to obtain more reliable evidence to support this hypothesis.

#### 4.2.4 Possible mechanisms for alleviating chemotherapy-related adverse reactions

This study also found that combination of thymosin α1 and chemotherapy may reduce incidence of various chemotherapy-related adverse reactions. This effect may be related to following mechanisms: First, by enhancing immune function, thymosin α1 may promote repair and regeneration of hematopoietic stem cells in bone marrow, thereby alleviating chemotherapy-induced myelosuppression (62); Second, improved immune status may enhance repair capacity of gastrointestinal mucosa, thereby mitigating direct damage caused by chemotherapeutic agents to digestive tract mucosa (63, 64); furthermore, thymosin α1 may reduce toxic effects of chemotherapeutic agents on the liver and peripheral nerves by regulating activity of drug-metabolizing enzymes or through antioxidant stress mechanisms (65). A study by Liu et al. also demonstrated that thymosin α1 can significantly reduce incidence of lymphocytopenia and pneumonia caused by chemoradiotherapy (66). This finding is of great significance for clinical practice, suggesting that thymosin α1 can serve as an adjunct to chemotherapy, enhancing efficacy while mitigating adverse reactions and improving patients’ treatment tolerance and quality of life.

### 4.3 Comparison with previous studies

Compared with previous studies, this study has following advantages: First, this is the first meta-analysis to systematically evaluate application of thymosin α1 in combination with chemotherapy for gastric cancer, filling a research gap in this field; second, the study included a large number of trials (20 RCTs) and a large sample size (1,704 patients), resulting in high statistical power; Third, outcome measures were comprehensive, covering multiple dimensions including efficacy, quality of life, immune function, tumor markers, inflammatory factors, and safety, thereby systematically evaluating overall effects of combination therapy; Fourth, a rigorous risk of bias assessment was conducted using RoB 2 tool to ensure quality of included RCTs was well-controlled; Fifth, the robustness and reliability of results were ensured through multidimensional analyses, including meta-regression, subgroup analysis, sensitivity analysis, publication bias assessment, TSA, and GRADE grading.

Furthermore, previous meta-analyses of thymosin α1 have primarily focused on its application in infectious diseases (such as severe pancreatitis and sepsis) or in other solid tumors (such as non-small cell lung cancer and liver cancer) (21, 67–71). This study focuses specifically on gastric cancer, providing dedicated evidence-based medical evidence for this patient population. Furthermore, this study included only RCTs, resulting in a higher level of evidence.

### 4.4 Limitations

However, this study also has certain limitations. First, all of the included studies were small-sample studies conducted in China, which may introduce regional and other biases, limiting generalizability of the findings to other populations and treatment settings; therefore, generalizability of the study conclusions requires further validation. Second, publication bias was observed for certain outcome measures (e.g., DCR); although the conclusions remained unchanged after correction using “trim-and-fill” method, these findings should still be interpreted with caution; at the same time, most studies had small sample sizes, resulting in insufficient statistical power, and the impact of selective reporting on other outcomes cannot yet be completely ruled out. Third, there is a high heterogeneity in outcomes such as immune function markers, tumor markers, and inflammatory factors. Although we have conducted a meta-regression analysis to extent feasible, we have not fully elucidated the sources of this heterogeneity. This variability may stem from combined effects of multiple factors, including differences in chemotherapy regimens, patient disease staging, baseline immune status, variations in the timing of blood sample collection for laboratory testing, and differences in the assay methods used across studies. Due to the limited number of studies and unavailability of detailed individual-level data, we were unable to conduct a systematic analysis of these factors. Fourth, most studies did not report long-term survival outcomes (such as OS and PFS), and it remains unclear whether thymosin α1 combined with chemotherapy translates into survival benefits; Fifth, some studies had issues with randomization or outcome measurement, resulting in limited study quality; furthermore, GRADE evidence quality assessment indicates that evidence quality for most outcome measures is low or very low, suggesting limited certainty in study conclusions; future high-quality research may alter the current findings.

Given above limitations, thymosin α1 cannot yet be widely recommended for routine gastric cancer treatment; its efficacy and safety require further validation through additional multicenter, international, and methodologically rigorous RCTs.

### 4.5 Future research directions

Future research should focus on following areas: First, conduct multicenter, large-sample, double-blind, placebo-controlled RCTs to provide higher-quality evidence-based medical evidence; second, extend follow-up period to clarify impact of thymosin α1 combined with chemotherapy on long-term survival outcomes; Third, explore the optimal administration regimen for thymosin α1, including optimization of dosage, frequency, and duration of treatment; Fourth, report more detailed individual patient data and conduct more in-depth subgroup analyses to identify subgroups that may benefit from combination therapy, and explore personalized treatment strategies based on biomarkers (such as baseline immune function status and PD-L1 expression levels); Fifth, to conduct an in-depth investigation into the mechanisms by which thymosin α1 enhances efficacy and reduces toxicity, particularly the molecular mechanisms underlying mitigation of chemotherapy-related toxicity; Sixth, given the widespread use of immune checkpoint inhibitors in gastric cancer treatment, the potential of combining thymosin α1 with immunotherapy warrants further exploration; Seventh, to conduct pharmacoeconomic evaluations to assess the cost-effectiveness of combination therapy.

## 5 Conclusion

Current evidence suggests that, compared with chemotherapy alone, thymosin α1 combined with chemotherapy may improve short-term clinical outcomes, enhance patients’ quality of life, boost immune function, reduce tumor marker levels, modulate inflammatory cytokine profile, and be associated with a lower incidence of chemotherapy-related adverse reactions. However, since the included studies were all small-sample studies conducted in China and of limited quality, the quality of evidence for most outcomes is low, and long-term benefits remain unclear. These conclusions require further validation through additional high-quality, multicenter, large-scale RCTs. Future research should focus on long-term survival benefits, optimization of optimal dosing regimen, identification of target patient populations, and in-depth exploration of underlying mechanisms of action.

## Data Availability

The original contributions presented in the study are included in the article/**Supplementary Material**. Further inquiries can be directed to the corresponding author.
