# Supplementary material for: Efficacy and safety of thymosin α1 combined with chemotherapy for gastric cancer: a systematic review and meta-analysis of randomized controlled trials
Source: Front Immunol. 2026 Sep 18;17:1884797. doi: 10.3389/fimmu.2026.1884797 (PMC13630780; doi:10.3389/fimmu.2026.1884797)
Supplement: Supplementary file 1 [file SupplementaryFile1.docx]

| Outcome and follow-up | Patients (studies), N | Relative effect (95% CI) | **Absolute effects (95% CI)** | | | Certainty |
| --- | --- | --- | --- | --- | --- | --- |
|  |  |  | **Control** | **Thymosin α1** | **Difference** |  |
| ORR | 1362 (16 RCTs) | **RR = 1.39** (1.26 to 1.53) | 490 per 1,000 | **681 per 1,000** (617 to 749) | **191 more per 1,000** (from 127 more to 260 more) | ⨁⨁⨁◯ Moderate^a^ |
| DCR | 1072 (13 RCTs) | **RR = 1.16** (1.10 to 1.24) | 795 per 1,000 | **923 per 1,000** (875 to 986) | **127 more per 1,000** (from 80 more to 191 more) | ⨁⨁◯◯ Low^a,b^ |
| KPS improvement rate | 270 (3 RCTs) | **RR = 2.01** (1.51 to 2.69) | 299 per 1,000 | **602 per 1,000** (452 to 805) | **302 more per 1,000** (from 153 more to 506 more) | ⨁⨁◯◯ Low^a,c^ |
| Gastrointestinal reactions | 662 (7 RCTs) | **RR = 0.63** (0.44 to 0.89) | 500 per 1,000 | **315 per 1,000** (220 to 445) | **185 fewer per 1,000** (from 280 fewer to 55 fewer) | ⨁◯◯◯ Very low^a,c,d^ |
| Myelosuppression | 414 (5 RCTs) | **RR = 0.60** (0.45 to 0.81) | 413 per 1,000 | **248 per 1,000** (186 to 334) | **165 fewer per 1,000** (from 227 fewer to 78 fewer) | ⨁⨁◯◯ Low^a,c^ |
| Neurotoxicity | 420 (4 RCTs) | **RR = 0.47** (0.31 to 0.69) | 276 per 1,000 | **130 per 1,000** (86 to 191) | **146 fewer per 1,000** (from 191 fewer to 86 fewer) | ⨁⨁◯◯ Low^a,c^ |
| Liver dysfunction | 532 (5 RCTs) | **RR = 0.54** (0.38 to 0.78) | 229 per 1,000 | **124 per 1,000** (87 to 179) | **105 fewer per 1,000** (from 142 fewer to 50 fewer) | ⨁⨁◯◯ Low^a,c^ |
| **CI:** confidence interval; **RR:** risk ratio | | | | | | |

a. Downgraded by one level: None of the studies mentioned the implementation of allocation concealment and blinding methods.

b. Downgrade by one level: Egger's test p < 0.05.

c. Downgraded by one level: Insufficient number of studies reporting outcomes, n < 10.

d. Downgraded by one level: 50% ≤ I² < 75%.

| Outcome and follow-up | Patients (studies), N | Relative effect (95% CI) | **Absolute effects (95% CI)** | | | Certainty |
| --- | --- | --- | --- | --- | --- | --- |
|  |  |  | **Control** | **Thymosin α1** | **Difference** |  |
| CD3^+^% | 629 (7 RCTs) | - | 0 | - | **7.73** (5.60 to 9.87) | ⨁◯◯◯ Very low^a,b,c^ |
| CD4^+^% | 1276 (14 RCTs) | - | 0 | - | **6.87** (5.37 to 8.36) | ⨁◯◯◯ Very low^a,e^ |
| CD8^+^% | 1174 (13 RCTs) | - | 0 | - | **-0.45** (-3.57 to 2.67) | ⨁◯◯◯ Very low^a,d,e^ |
| CD4^+^/CD8^+^ | 956 (11 RCTs) | - | 0 | - | **0.42** (0.24 to 0.61) | ⨁◯◯◯ Very low^a,e^ |
| NK | 658 (6 RCTs) | - | 0 | - | **4.73** (2.15 to 7.3) | ⨁◯◯◯ Very low^a,c,e^ |
| KPS | 402 (5 RCTs) | - | 0 | - | **8.27** (4.21 to 12.32) | ⨁◯◯◯ Very low^a,c,e^ |
| CEA | 287 (3 RCTs) | - | 0 | - | **-5.48** (-10.58 to -0.39) | ⨁◯◯◯ Very low^a,c,e^ |
| CA199 | 185 (2 RCTs) | - | 0 | - | **-9.06** (-12.5 to -5.61) | ⨁⨁◯◯ Low^a,c^ |
| MMP-2 | 372 (4 RCTs) | - | 0 | - | **-25.6** (-43.56 to -7.64) | ⨁◯◯◯ Very low^a,c,e^ |
| MMP-9 | 372 (4 RCTs) | - | 0 | - | **-50.63** (-62.98 to -38.27) | ⨁◯◯◯ Very low^a,c,e^ |
| TNF-α | 178 (2 RCTs) | - | 0 | - | **1.67** (0.94 to 2.41) | ⨁◯◯◯ Very low^a,c,e^ |
| IFN-γ | 178 (2 RCTs) | - | 0 | - | **3.33** (2.19 to 4.47) | ⨁◯◯◯ Very low^a,b,c^ |
| IL-4 | 178 (2 RCTs) | - | 0 | - | **-2.12** (-2.41 to -1.82) | ⨁⨁◯◯ Low^a,c^ |
| IL-10 | 178 (2 RCTs) | - | 0 | - | **-1.01** (-1.82 to -0.21) | ⨁◯◯◯ Very low^a,c,e^ |
| **CI:** confidence interval; **MD:** mean difference | | | | | | |

a. Downgraded by one level: None of the studies mentioned the implementation of allocation concealment and blinding methods.

b. Downgraded by one level: 50% ≤ I² < 75%.

c. Downgraded by one level: Insufficient number of studies reporting outcomes, n < 10.

d. Downgrade by one level: The 95% confidence interval for the pooled effect size spans the boundary of non-significance.

b. Downgraded by two levels: I² > 75%.
