# Supplementary material for: Efficacy and safety of thymosin α1 combined with chemotherapy for gastric cancer: a systematic review and meta-analysis of randomized controlled trials
Source: Front Immunol. 2026 Sep 18;17:1884797. doi: 10.3389/fimmu.2026.1884797 (PMC13630780; doi:10.3389/fimmu.2026.1884797)
Supplement: Supplementary file 2 [file SupplementaryFile2.docx]

**Figure S1a:** Forest plot of ORR (patient age).

**Figure S1b:** Forest plot of ORR (dosing frequency).

**Figure S1c:** Forest plot of ORR (total dose).

**Figure S1:** Forest plot of ORR subgroup analysis.

**Figure S2a:** Forest plot of DCR (patient age).

**Figure S2b:** Forest plot of DCR (dosing frequency).

**Figure S2c:** Forest plot of DCR (total dose).

**Figure S2:** Forest plot of DCR subgroup analysis.

**Figure S3a:** Forest plot of CD3⁺ T% (patient age).

**Figure S3b:** Forest plot of CD3⁺ T% (dosing frequency).

**Figure S3c:** Forest plot of CD3⁺ T% (total dose).

**Figure S3:** Forest plot of CD3⁺ T% subgroup analysis.

**Figure S4a:** Forest plot of CD4⁺ T% (patient age).

**Figure S4b:** Forest plot of CD4⁺ T% (dosing frequency).

**Figure S4c:** Forest plot of CD4⁺ T% (total dose).

**Figure S4:** Forest plot of CD4⁺ T% subgroup analysis.

**Figure S5a:** Forest plot of CD4⁺/CD8⁺ ratio (patient age).

**Figure S5b:** Forest plot of CD4⁺/CD8⁺ ratio (dosing frequency).

**Figure S5c:** Forest plot of CD4⁺/CD8⁺ ratio (total dose).

**Figure S5:** Forest plot of CD4⁺/CD8⁺ ratio subgroup analysis.

**Figure S6a:** Sensitivity analyses of DCR.

**Figure S6b:** Sensitivity analyses of KPS score.

**Figure S6c:** Sensitivity analyses of KPS improvement rate.

**Figure S6d:** Sensitivity analyses of CD3⁺%.

**Figure S6e:** Sensitivity analyses of CD4⁺%.

**Figure S6f:** Sensitivity analyses of NK.

**Figure S6g:** Sensitivity analyses of CEA.

**Figure S6h:** Sensitivity analyses of CA199.

**Figure S6i:** Sensitivity analyses of MMP-2.

**Figure S6j:** Sensitivity analyses of MMP-9.

**Figure S6k:** Sensitivity analyses of myelosuppression.

**Figure S6l:** Sensitivity analyses of neurotoxicity.

**Figure S6:** Sensitivity analyses of other outcomes.

**Figure S7a:** Funnel plot of CD4⁺%.

**Figure S7b:** Funnel plot of CD8⁺%.

**Figure S7c:** Funnel plot of CD4⁺/CD8⁺ ratio.

**Figure S7:** Funnel plot of other outcomes.

**Figure S8a:** TSA result of DCR.

**Figure S8b:** TSA result of myelosuppression.

**Figure S8:** TSA results of other outcomes.
